# Evolution of left ventricular function among subjects with ST-elevation myocardial infarction after percutaneous coronary intervention

**DOI:** 10.1186/s12872-020-01540-y

**Published:** 2020-06-29

**Authors:** Ulrika Pahlm, Ellen Ostenfeld, Felicia Seemann, Henrik Engblom, David Erlinge, Einar Heiberg, Håkan Arheden, Marcus Carlsson

**Affiliations:** 1 Clinical Physiology, Department of Clinical Sciences Lund, Lund University, Skane University Hospital, Lund, Sweden; 2 Department of Emergency Medicine, Skane University Hospital, Lund, Sweden; 3 Department of Biomedical Engineering, Faculty of Engineering, Lund University, Lund, Sweden; 4 Cardiology, Department of Clinical Sciences Lund, Lund University, Skane University Hospital, Lund, Sweden; 5 Wallenberg Center for Molecular Medicine, Lund University, Lund, Sweden

**Keywords:** Atrioventricular plane displacement, STEMI, Left ventricular function, Longitudinal function, Radial function, Wall thickening

## Abstract

**Background:**

Atrioventricular plane displacement (AVPD) reflects longitudinal left ventricular (LV) systolic function, and wall thickening (WT) regional radial LV function. The temporal evolution of these measures after STEMI with CMR has not been evaluated. We aimed to investigate how AVPD and WT are affected globally and regionally from the sub-acute to the chronic phase after ST-elevation myocardial infarction (STEMI).

**Methods:**

Healthy volunteers without cardiovascular disease and medication (controls, *n* = 20) and patients from the CHILL-MI study (NCT01379261) prospectively underwent magnetic resonance imaging (MRI) 2–6 days and 6 months after STEMI (*n* = 77). CHILL-MI randomized STEMI-patients to cooling therapy initiated before reperfusion or standard of care. AVPD was measured at six points in three long axis cine images and wall thickening in short axis cine images. Infarction was quantified using late gadolinium enhancement (LGE) and used to define infarct and remote segments.

**Results:**

There were no difference in AVPD either at acute or chronic phase (*p* = 0.90 and *p* = 0.40) or WT (*p* = 0.85 and *p* = 0.99) between patients randomized to cooling therapy and standard of care. Therefore, the results are presented for the pooled cohort. Global AVPD was decreased in both the sub-acute (12 ± 2 mm, *p* < 0.001) and the chronic phase (13 ± 2 mm, p < 0.001) compared to controls (15 ± 2 mm) with a partial recovery of AVPD (p < 0.001) in the chronic phase. Patients with left anterior descending (LAD) and right coronary artery (RCA) infarcts had decreased AVPD in the chronic phase in both infarcted and remote segments. Mean WT was decreased in patients with LAD infarction both in the sub-acute and the chronic phase in both infarcted and remote segments. The decrease in WT in patients with RCA and left circumflex (LCx) infarcts was more affected in the infarcted segments, especially in the chronic phase.

**Conclusion:**

AVPD was a global rather than regional marker of cardiac function in this STEMI study and this may explain the prognostic importance of local measurements of mitral annular plane systolic excursion (MAPSE). The decrease in WT in remote myocardium even in the chronic phase needs to be taken into consideration when combining functional measurements with infarct quantification for diagnosis of post-ischemic stunning and hibernation.

## Background

Myocardial infarction (MI) is a major cause of heart failure as well as death [1]. Infarct size and microvascular obstruction can be localized and quantified by late gadolinium enhancement from cardiovascular magnetic resonance (CMR) imaging and are predictors of survival in patients with ST-elevation MI (STEMI) [2, 3]. Furthermore, reduced left ventricular (LV) function is an independent predictor of mortality [4, 5]. Regional LV function decreases in the MI territory and this can be visualized on both echocardiography and CMR imaging. Recognition of decreased regional function caused by MI is typically done by visual assessment of radial wall thickening (WT). A mismatch between MI on late gadolinium enhancement and WT is used to detect post-ischemic stunning and hibernation. Methods for quantifying regional functional change instead of only visualizing them are increasingly used. Global longitudinal LV shortening measured as global longitudinal strain (GLS) can be used to detect MI [6], and has prognostic value superior to ejection fraction (EF) [7]. Longitudinal LV systolic shortening is also reflected by the atrioventricular plane displacement (AVPD) [8] which has been shown to be the main contributor to LV stroke volume in healthy individuals [9] and in patients with STEMI, both in the sub-acute [10] and the chronic phases [11]. GLS and AVPD differs as GLS quantifies myocardial shortening in the long-axis direction and the latter measure the summed effect of this contraction on the AV-plane. Of note, the AVPD links systolic ventricular contraction to systolic atrial filling and is therefore a measure of atrio-ventricular interaction [12] [9]. Furthermore, AVPD measured by either echocardiography [13] or CMR [14, 15] provides strong prognostic information on major adverse cardiovascular events including mortality.

We have previously shown that AVPD is decreased in all LV segments within the first week of STEMI, even in segments remote from the infarcted area [10]. This suggests that distinguishing infarcted from non-infarcted segments is difficult using regional longitudinal measures. It is not known if the global decrease in AVPD measured with CMR persists in the chronic phase.

Regional radial LV function is considered to be reduced mainly in infarcted regions after MI [16–18]. However, lack of reference values and high inter-vendor variability in echocardiography still need to be addressed before recommending quantitative measurements [19]. Standard cine CMR imaging has the advantage of better delineation of the endocardial, and in particular the epicardial border than echocardiography, and quantitative regional WT may therefore be more suitable with this technique.

However, the evolution of LV longitudinal and radial function in patients after STEMI has not been fully explored with CMR. Thus, we aimed to investigate the evolution of longitudinal LV function, measured as AVPD, and radial function, measured as WT, globally and regionally from the sub-acute (2–6 days) to the chronic phase (6 months) after STEMI.

## Methods

### Study population

Patients from the international multicenter cardioprotection study CHILL-MI (NCT01379261) [20] were considered for this sub-study. Patients who presented with chest pain lasting for less than 6 h, were over 18 years old and had a first STEMI were included in CHILL-MI. Patients were randomized to cooling or no cooling therapy and underwent percutaneous coronary intervention (PCI) with successful reperfusion of the occluded vessel. Patients included in the present study prospectively underwent CMR imaging 2–6 days after STEMI (*sub-acute phase*) and had follow-up CMR imaging after 6 months (*chronic phase*). Twenty healthy, age-matched controls from a previously published study [10] were included for comparison. The control group were healthy volunteers without any history of cardiovascular disease and without any cardiovascular medication, they had a normal ECG and blood pressure < 140/90 mmHg. Informed consent was obtained from all subjects in the original study. The Regional Ethical Review Board in Lund, Sweden, approved the CHILL-MI study and permission from local ethical review boards was granted at each center.

### CMR image acquisition

The imaging protocol has been published previously [10]. In short, CMR was performed at multiple clinical centers using Siemens, Philips or General Electric magnetic resonance imaging (MRI) scanners. Images were acquired with patients in the supine position, at end-expiratory breath-hold with retrospective ECG gating. Following administration of 0.2 mmol/kg gadolinium, steady-state-free precession cine short-axis, and 2-, 3- and 4-chamber long-axis images were acquired. Twenty to 30 timeframes per cardiac cycle were obtained. Fifteen to 20 min after injection of the gadolinium contrast agent, late gadolinium-enhanced images were obtained. Typical spatial resolution was 1.5x1.5x8 mm.

### CMR analysis

Image analysis was performed using Segment version 2.0 (http://segment.heiberg.se) [21] as previously described [22, 23]. In short, readers (HE, MC, HA) in a core lab (Imacor AB, Lund, Sweden) delineated the endo- and epicardium in the short-axis cine images in end-diastole (ED) and end-systole (ES). Stroke volume, end-systolic volume (ESV), end-diastolic volume (EDV), left ventricular mass (LVM), epicardial surface area and ejection fraction (EF) were calculated.

**Fig. 1:**
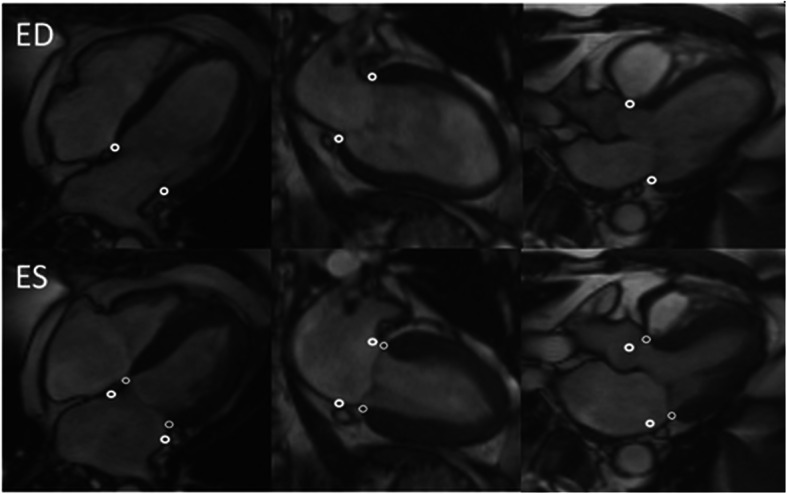
Determination of atrioventricular plane displacement (AVPD). Long-axis cine images with the AVPD locations marked in end-diastole (ED) with thick white circles and in end-systole (ES) with thin white circles. Right column shows the 4-chamber, middle column the 2-chamber and left column the 3-chamber images. The AVPD between ED and ES was measured perpendicular to the AV-plane in ED.

The atrioventricular (AV) plane was identified in the anterior, anteroseptal, inferoseptal, inferior, inferolateral, and anterolateral segments of the LV at ED and ES using long-axis steady-state-free precession images (Fig. 1). Mean AVPD, as well as AVPD for each segment were calculated. The stroke volume generated by AVPD was determined by multiplying the mean AVPD (cm) with the LV-short axis epicardial area (cm^2^) as previously described and validated [9].

To determine WT for each segment we used mid-ventricular short-axis images. Wall thickening is the percent change of LV segment thickness between ED and ES calculated as WT =100* (ES wall thickness – ED wall thickness)/ ED wall thickness. We chose mid-ventricular images to reduce the effect of longitudinal movement in the images. Infarct size (IS) was quantified in late gadolinium enhancement images after manual delineation of the endocardial and epicardial borders using a validated semi-automatic algorithm with manual corrections [22].

### Infarcted, adjacent and remote myocardium

Infarcted segments were defined from the AHA 17-segment model and presence of infarction in these segments was verified in all patients using mid-ventricular late gadolinium enhancement images. Absence of infarction in remote segments was also verified in these late gadolinium enhancement images. Thus, anterior and anteroseptal segments were considered infarcted in patients with LAD occlusion, inferoseptal and anterolateral segments adjacent, and inferior and inferolateral segments remote. In patients with RCA occlusion, inferoseptal and inferior segments were considered infarcted, anteroseptal and inferolateral adjacent, and anterior and anterolateral remote. In patients with LCx infarct, inferolateral and anterolateral segments were considered infarcted, inferior and anterior adjacent, and anteroseptal and inferoseptal remote.

### Statistical analysis

Statistical analysis was performed using Microsoft Excel 2010 and Prism 5.0 (GraphPad Software Inc.). Continuous variables are presented as mean ± SD. Paired Student’s t test was used to compare the sub-acute and chronic phase measurements. Un-paired t-test was used to compare patient and control measurements. Pearson’s correlation analysis was used for assessing the relationship between IS and EF. Results with a *p*-value < 0.05 were considered statistically significant.

## Results

### Subject characteristics

Seventy-seven patients from CHILL-MI underwent sub-acute and chronic CMR imaging. Late gadolinium enhancement images from 3 patients in the chronic phase were missing, and they were excluded from IS analysis. Characteristics of patients and healthy controls are presented in Table 1.

**Table 1:** Subject characteristics

	Sub-acute phase (*n* = 77)	Chronic phase (*n* = 77)	Controls (*n* = 20)
Age (years)	58 ± 10		62 ± 11
Sex, men/women (%)	88/12 ***		60/40
Heart rate (beats per minute)	69 ± 11 *	61 ± 8 †††	62 ± 7
Ejection fraction (%)	48 ± 8 ***	52 ± 9 *** †††	60 ± 5
Infarct size (%)	17 ± 9 ***	10 ± 6 *** †††	–
End-diastolic volume (ml)	179 ± 36	189 ± 42 ** †††	163 ± 36
End-systolic volume (ml)	94 ± 31 ***	92 ± 34 ***	66 ± 20
Stroke volume (ml)	85 ± 16 ***	97 ± 22 †††	97 ± 20
Left ventricular mass (g)	127 ± 26 *	112 ± 26 †††	112 ± 31
Mean AVPD (mm)	12 ± 2 ***	13 ± 2 *** †††	15 ± 2
AVPD contribution to stroke volume (%)	59 ± 9 *	58 ± 9 **	64 ± 8

*AVPD* atrioventricular plane displacement

Differences between patients and controls; * = *p* < 0.05 ** = *p* < 0.01 *** = *p* < 0.001

Differences between patients at 2–6 days and 6 months: ††† = *p* < 0.001

There was a larger proportion of men in the patient group than in the control group, but ages were similar. The slightly increased heart rate seen in the sub-acute phase compared to controls was normalized in the chronic phase. End-diastolic volume was larger in the chronic phase than in the sub-acute phase showing post-MI remodeling. End-diastolic volume was also larger than in controls. Stroke volume increased from the sub-acute to the chronic phase to the same level as controls. The increased LV mass seen in the sub-acute phase decreased in the chronic phase and then did not differ from controls. Infarct size decreased from the sub-acute to the chronic phase for the entire patient population (Table 1), and when dividing patients into subgroups according to culprit artery (Table 2).

**Table 2:** Evolution of myocardial infarction size and left ventricular function

Culprit vessel	Infarct size %		Ejection fraction % Controls 60 ± 5		Mean AVPD (mm) Controls 15 ± 2	
	Sub-acute	Chronic	Sub-acute	Chronic	Sub-acute	Chronic
All (*n* = 77)	17 ± 10	10 ± 6 †††	48 ± 8***	52 ± 9***,†††	12 ± 2***	13 ± 2***,†††
LAD (*n* = 28)	24 ± 8	14 ± 6 †††	45 ± 9***	49 ± 11***,†	11 ± 2***	13 ± 2***,†††
RCA (*n* = 39)	14 ± 8	9 ± 5 †††	51 ± 7**	54 ± 8**,†	12 ± 2***	13 ± 3***,††
LCx (n = 10)	13 ± 5	7 ± 3 ††	49 ± 7***	55 ± 9††	13 ± 2**	14 ± 3

Left anterior descending = LAD. Right coronary artery = RCA. Left circumflex = LCx. AVPD = atrioventricular plane displacement

Comparison between patients and controls; ** = *p* < 0.01 *** = *p* < 0.001

Comparison between sub-acute and chronic phases: † = *p* < 0.04 †† = *p* < 0.01 ††† = p < 0.001

**Fig. 2:**
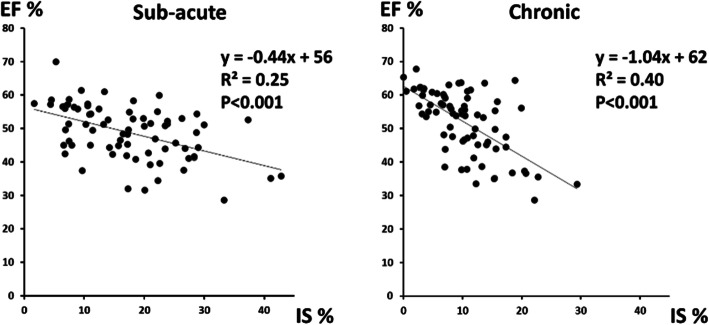
Relationship between ejection fraction (EF) and infarct size (IS) in the sub-acute phase (left panel) and the chronic phase (right panel) after STEMI. Infarct size is expressed as the percent infarcted myocardium of total left ventricular mass

EF increased in patients from the sub-acute to the chronic phase but remained decreased compared to controls except in patients with LCx infarction. The negative correlation between infarct size and EF in the sub-acute phase (r = − 0.50) (Fig. 2) was slightly higher in the chronic phase (r = − 0.63).

### Global longitudinal function

AVPD did not differ between patients with or without treatment with cooling, neither in the sub-acute (*p* = 0.9) nor in the chronic phase (*p* = 0.4). Mean AVPD was decreased in patients compared to controls both in the sub-acute and the chronic phases, and there was a partial recovery from the sub-acute to the chronic phase (Table 2). In LAD and RCA patients mean AVPD was decreased in both the sub-acute and chronic phases. In LCx patients, however, AVPD was decreased only in the subacute phase (Table 2).

### Segmental longitudinal function

**Fig. 3:**
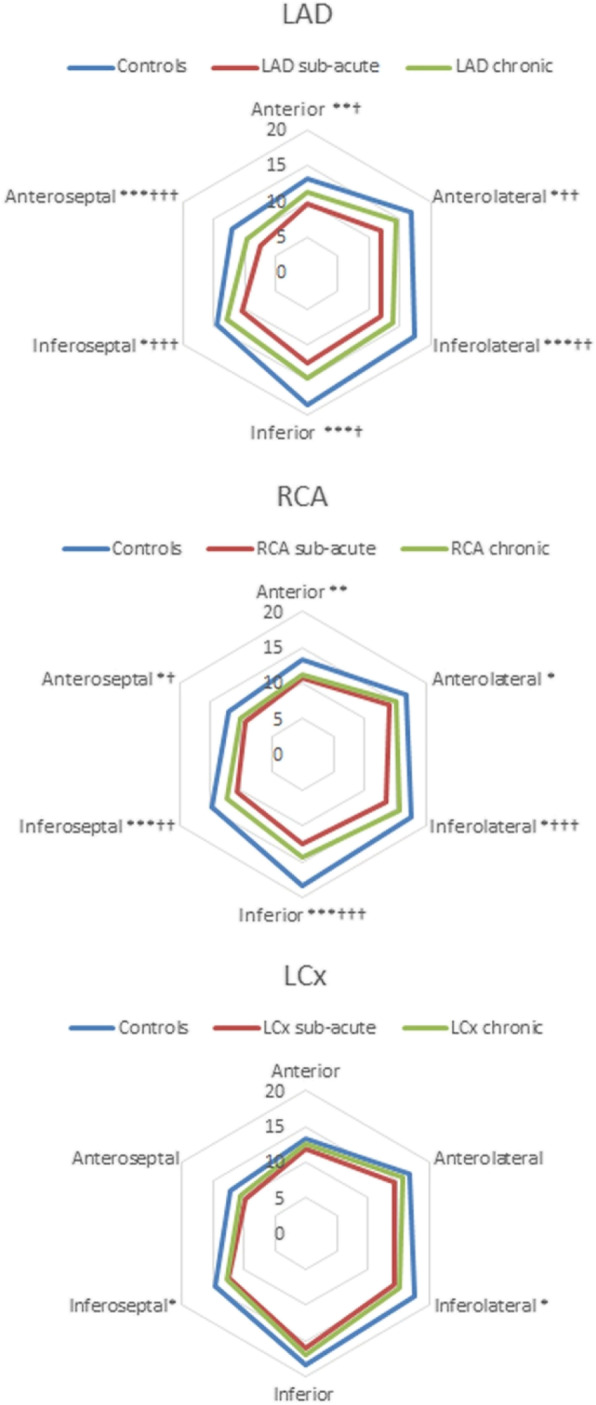
Longitudinal function. Regional atrioventricular plane displacement (AVPD, in millimeters) demonstrated for each culprit vessel. Upper panel presents results for controls and patients with infarction in left anterior descending coronary artery (LAD) territory, middle panel controls and patients with infarction in the right coronary (RCA) and lower panel present results for controls and patients with infarction in the left circumflex (LCx) coronary artery territory. Bold blue lines represent controls, red lines patients 2–6 days after ST-elevation myocardial infarction and green lines patients 6 months after ST-elevation myocardial infarction. Comparison between chronic phase and controls: * = *p* < 0.05. ** = p < 0.01 *** = *p* < 0.001. Comparison between sub-acute and chronic phase: † = *p* < .05 †† = p < 0.01 ††† = p < 0.001

**Fig. 4:**
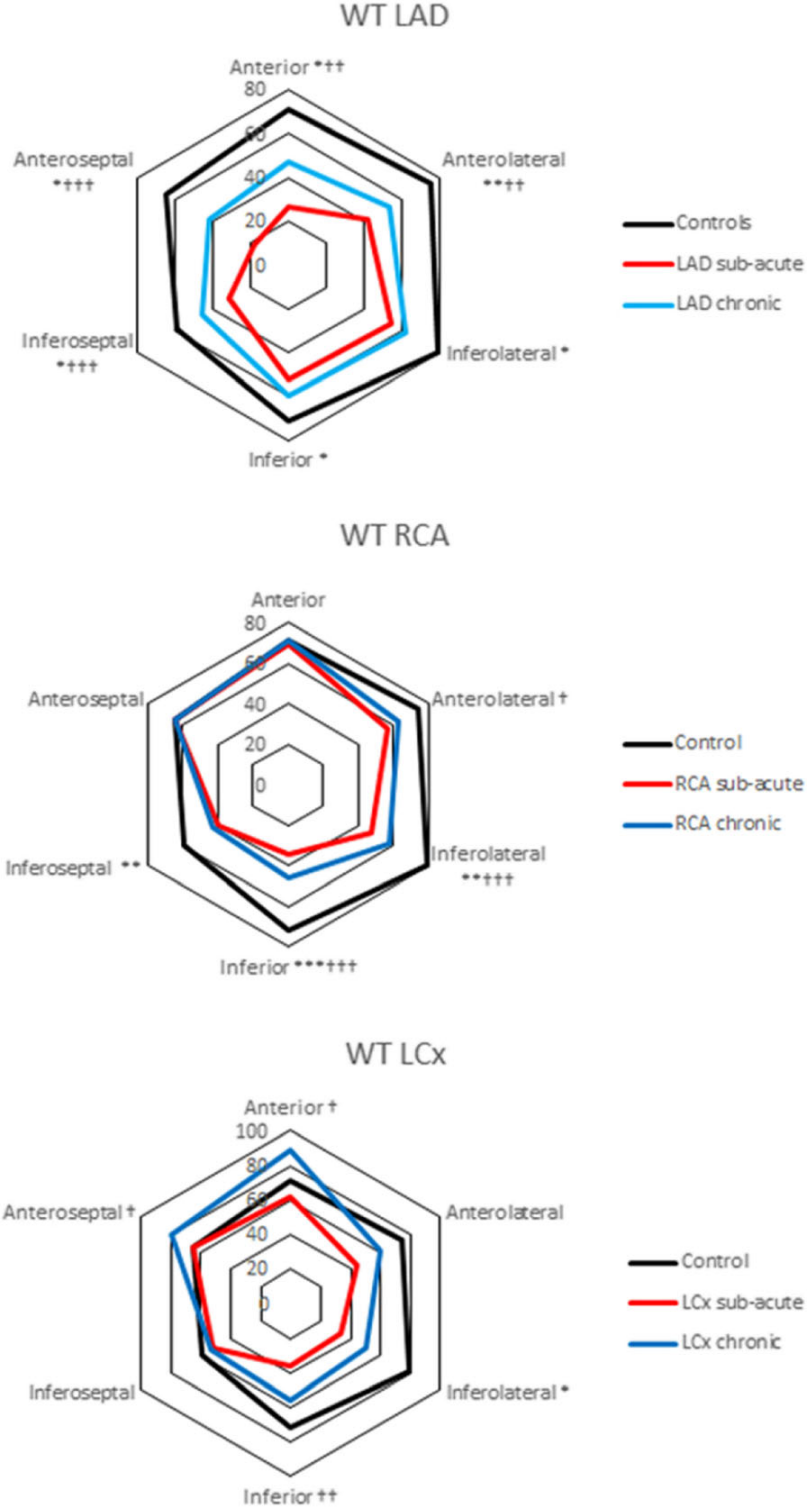
Radial function. Wall thickening (WT) in percent (%) between end-diastole and end-systole demonstrated for each culprit vessel. Upper panel presents results for controls and patients with infarction in left anterior descending coronary artery (LAD) territory, middle panel controls and patients with infarction in the right coronary (RCA) and lower panel presents results for controls and patients with infarction in the left circumflex (LCx) coronary artery territory. Black lines represent controls, red lines patients in the sub-acute phase after ST-elevation myocardial infarction and blue lines patients in the chronic phase after ST-elevation myocardial infarction. Comparison between chronic phase and controls: * = *p* < 0.05. ** = p < 0.01 *** = *p* < 0.001. Comparison between sub-acute and chronic phase † = *p* < .05 †† = p < 0.01 ††† = p < 0.001

Figure 3 and Table 3 show results from regional AVPD analysis of patients divided into sub-groups according to culprit artery and controls. There was a partial recovery in regional AVPD in patients with LAD and RCA infarcts. However, longitudinal function remained decreased in the chronic phase in both infarcted and remote segments compared to controls (Fig. 3, upper and middle panel). In patients with LCx infarcts, AVPD was decreased in all but one remote segment in the sub-acute phase. This decrease remained significant only in 2 segments in the chronic phase (Fig. 4 lower panel, Table 3). This sub-group was small (*n* = 10) and therefore had lower statistical power which may explain why these patients had decreased AVPD in fewer segments.

**Table 3:** Segmental atrioventricular plane displacement (mm)

Segment	Anterior	Anteroseptal	Inferoseptal	Inferior	Inferolateral	Anterolateral	Mean AVPD
Controls (n = 28)	13 ± 2	12 ± 2	15 ± 2	18 ± 2	18 ± 2	17 ± 2	15 ± 2
LAD Sub-acute (n = 28)	I 10 ± 3***	I 8 ± 2***	A 11 ± 3***	R 13 ± 3***	R 12 ± 4***	A 12 ± 2***	11 ± 2***
LAD Chronic (n = 28)	I 11 ± 2**,†	I 10 ± 2***,†††	A 13 ± 3*,†††	R 15 ± 3***,†	R 14 ± 3***,††	A 15 ± 3*,††	13 ± 2***,††
RCA Sub-acute (n = 39)	R 11 ± 3**	A 9 ± 2***	I 11 ± 2***	I 13 ± 3***	A 14 ± 3***	R 14 ± 2***	12 ± 2***
RCA Chronic (n = 39)	R 11 ± 3**	A 10 ± 3*,†	I 12 ± 3***,††	I 15 ± 3***,†††	A 16 ± 3*,†††	R 15 ± 3*	13 ± 3**,††
LCx Sub-acute (n = 10)	A 12 ± 2	R 10 ± 2*	R 13 ± 2**	A 16 ± 3*	I 14 ± 2**	I 14 ± 3*	13 ± 2**
LCx Chronic (n = 10)	A 13 ± 2	R 11 ± 3	R 13 ± 3*	A 17 ± 4	I 15 ± 3*	I 16 ± 3	14 ± 3

AVPD = atrioventricular plane displacement (mean ± SD; mm). LAD = left anterior descending

RCA = right coronary artery. LCx = left circumflex artery

I = infarcted. A = adjacent. R = remote

Comparison between patients and controls * = p < 0.05 ** = p < 0.01 *** = p < 0.001

Comparison between sub-acute and chronic † = *p* < 0.05 †† = p < 0.01 ††† = p < 0.001

### Longitudinal contribution to stroke volume

The relative contribution of AVPD (%) to stroke volume was decreased in patients in the sub-acute (59 ± 9%, *p* < 0.05) and chronic phases (58 ± 9%, *p* < 0.01) compared to controls (64 ± 8%, Table 1).

### Segmental radial function

There was no difference in WT in the patient group receiving cooling therapy compared to the group that did not (*p* = 0.85 for subacute and *p* = 0.99 for chronic phase).

Figure 4 and Table 4 show results from segmental WT analysis of controls and patients divided into sub-groups according to culprit artery. Mean WT was decreased in all LV segments in patients with LAD infarction in the sub-acute and chronic phases after STEMI compared to controls. The decrease was most prominent in the anterolateral, anterior anteroseptal, and inferoseptal segments. Partial recovery was seen in these segments in the chronic phase.

**Table 4:** Segmental wall thickening (%) in the sub-acute and chronic phases after STEMI

Segment	Anterior	Anteroseptal	Inferoseptal	Inferior	Inferolateral	Anterolateral
Controls (n = 20)	71 ± 25	65 ± 22	59 ± 18	72 ± 22	79 ± 30	75 ± 25
LAD Sub-acute (n = 28)	I 27 ± 17***	I 18 ± 15***	A 31 ± 15***	R 53 ± 17**	R 54 ± 23**	A 42 ± 20***
LAD Chronic (n = 28)	I 47 ± 34*,††	I 42 ± 27*,†††	A 46 ± 18*,†††	R 60 ± 15*	R 62 ± 16*	A 53 ± 24**,††
RCA Sub-acute (n = 39)	R 70 ± 21	A 64 ± 19	I 40 ± 13***	I 34 ± 18***	A 47 ± 21***	R 56 ± 21**
RCA Chronic (n = 39)	R 71 ± 20	A 64 ± 20	I 43 ± 18**	I 46 ± 23***,†††	A 58 ± 24**,†††	R 63 ± 20†
LCx Sub-acute (n = 10)	A 63 ± 21	R 63 ± 12	R 51 ± 15	A 37 ± 20***	I 36 ± 20***	I 46 ± 19**
LCx Chronic (*n* = 9)	A 89 ± 21†	R 80 ± 11†	R 54 ± 13	A 56 ± 14††	I 51 ± 22*	I 60 ± 21

LAD = left anterior descending RCA = right coronary artery. LCx = left circumflex artery

I = infarcted. A = adjacent. R = remote

Comparison between patients and controls * = p < 0.05 ** = *p* < 0.01 *** = *p* < 0.001

Comparison between sub-acute and chronic phase † = p < 0.05 †† = *p* < 0.01††† p = < 0.001

Patients with RCA infarcts had decreased WT in inferoseptal, inferior, inferolateral, and anterolateral, LV segments in the sub-acute phase with recovery of function in the anterolateral segment and partial recovery in inferolateral and inferior segments.

Patients with LCx infarcts had decreased WT in the inferior, inferolateral and anterolateral segments in the sub-acute phase. One patient in this group did not have short axis CMR images in the chronic phase and was therefore excluded from WT analysis in that phase. WT remained decreased only in the inferolateral segment.

## Discussion

This study has shown that global longitudinal function, measured as AVPD, is decreased in the sub-acute and chronic phases after STEMI whereas stroke volume increased from the sub-acute phase to the chronic phase to the same level as in controls. Regional AVPD was affected in both infarcted and remote areas in both the sub-acute and chronic phases. Wall thickening was also globally affected, particularly in LAD-infarcts but had more prominent differences between remote and infarcted walls in RCA and LCx-infarcts. The more global effect on regional function in LAD infarcts is probably due to the larger infarct size and possibly LV remodeling. Measures of regional longitudinal function will thus have limited accuracy for localizing infarction, and simple cut-off values for WT may also be unreliable. This information is also important when regional function is used in combination with LGE to identify post-ischemic stunning and hibernation.

### Regional and global longitudinal function

The magnitude of AVPD in the present study using CMR imaging is similar to that reported by Støylen et al. using echocardiography [24]. They reported that healthy controls had a mitral annular excursion of 16 mm compared to the 15 mm AVPD found in our study. Their controls were younger (average 36 years) than the controls in this study (average 62 years) and this could explain the possible, slight difference between the study results as AVPD is suggested to decrease with age [25]. Another explanation may be intermodality differences in assessment of AVPD between echocardiography and CMR imaging. Both Støylen et al. and our study show mean longitudinal AVPD of 12 mm sub-acutely after MI. The AVPD contribution to stroke volume is a measure linking LV systolic function with concomitant atrial filling. The magnitude of the descent of the AV-plane during systole is multiplied by the LV epicardial surface area that is bordering the atrium and thus the resulting volume is the same volume that the LV aspirates from the pulmonary veins into the left atrium [9]. We found a small decrease in AV-plane contribution to stroke volume in both the acute and chronic phase after STEMI compared to controls, in line with our previous findings. Thus, patients with STEMI has lower systolic atrial filling equivalent to a decrease in left atrial reservoir function, compared to controls.

Our findings suggest that assessment of longitudinal LV function using AVPD with CMR fails to differentiate between infarcted and non-infarcted myocardium in the subacute and chronic phases after STEMI. This may seem to be in contrast to results reported by Cimino et al. [26] who found significantly lower regional longitudinal strain in infarcted than in remote LV segments by 2D speckle-tracking echocardiography. We have previously shown a significant difference in longitudinal strain in infarct patients with MR velocity-encoding strain [27]. However, the diagnostic accuracy to detect an MI by longitudinal strain was limited with a sensitivity of 70% and specificity of 72% in an experimental animal model of infarction [17]. Rosendahl et al. showed a 64% sensitivity of echocardiography-based longitudinal strain to detect an MI [28]. The decreased global longitudinal function after STEMI seen in our study can help explain why global measures such as global longitudinal strain (GLS) can be used to detect the presence of MI. In a meta-analysis, Diao et al. [6] showed that the diagnostic accuracy of GLS to detect the presence of large MIs (> 12%) using echocardiography has a sensitivity of 77% and a specificity of 86%. However, to identify the vessel territory – and not only presence of infarction by using longitudinal measures is not possible, according to our results. Our findings are thus in line with these earlier results from regional longitudinal strain from echocardiography.

### Explanations for global decrease in AVPD after MI

The reasons for a global effect on AVPD by a regional ischemic injury has been proposed by Støylen [27] to be due to the anatomical fact that all four cardiac valves are joined in the *anulus fibrosus* and that this prohibits large regional differences in movement between, e.g., the anterior and inferior parts of the AV-plane. Furthermore, Støylen hypothesized that a decrease in myocardial shortening by an ischemic injury will decrease the stretch on surrounding myocardium and may thus decrease the load and increase the movement by surrounding myocardium, seen as hypercontractility in surrounding myocardium and resulting in similar AVPD in the ischemic and remote walls [29]. Another explanation is that remote myocardium is not unaffected by STEMI. Inflammatory cells that affect mitochondrial proteins [30] and expansion of extra-cellular volume have been found in remote myocardium [31]. Measurements of extra-cellular volume were, however, not available in our study. Finally, the use of medication after a STEMI such as betablockers may affect the myocardium globally.

### Radial function

In LAD patients, WT was decreased in the subacute and chronic phases in both infarcted, adjacent and remote segments, and the decrease was more pronounced in the infarcted and adjacent segments in the sub-acute phase. However, in the chronic phase the decrease in WT was similar in all segments. In contrast, both RCA and LCx patients had more localized decreased WT in the infarcted and adjacent segments. The definition of infarcted, adjacent and remote segments in this study is taken from the AHA 17-segment model. However, some patients with RCA infarct have a basal anterolateral infarction which means that midventricular anterolateral segment may be adjacent and not remote. This could explain why there is a decreased WT in this segment in RCA patients. Decreased WT in the remote myocardium has been shown to be a marker of long-term remodeling after a STEMI [32]. The challenge when using WT to identify MI location is thus both that the change of radial thickness is quite small and can be difficult to quantify visually and that both infarcted and remote myocardium have decreased WT particularly in large LAD infarcts. Furthermore, Everaars et al. showed that strain from tagging CMR was superior to WT when identifying infarcted and remote myocardium [33].

### Time course of LV function after MI

The time it takes for the LV to recover after MI is debated. Ingul et al. studied the recovery of LV function in 31 patients the first week after STEMI [34] and found that most of the systolic function was regained within the first 2 days. Engblom et al. followed 22 first-time STEMI patients over a year with CMR imaging and found an increase in radial WT between day 1 and 7 but also a further increase at later follow up [18]. Lately, Baron et al. found that GLS, measured by echocardiography, 1 year after MI was improved compared to baseline in patients with normal EF [35]. Further Hassell et al. has presented that LV remodeling is an ongoing process for at least 2 years after STEMI [32]. In our study, sub-acute CMR imaging was performed 2–6 days after STEMI and it is likely that LV function had partially recovered. As such, we found that there is a significant improvement of both longitudinal and radial function between the subacute and chronic phases, although the function did not reach the level of the controls.

### Limitations

The patient population in this study consists of a cooling group and a non-cooling group and the cooling therapy may have influenced the results. Albeit, we found no difference in AVPD or WT between these groups.

## Conclusions

AVPD was a global rather than regional marker of cardiac function in this STEMI study and this may explain the prognostic importance of local measurements of mitral annular plane systolic excursion (MAPSE). The decrease in WT in remote myocardium even in the chronic phase needs to be taken into consideration when combining functional measurements with infarct quantification for diagnosis of post-ischemic stunning and hibernation.

## Data Availability

The datasets generated and analysed during the current study are not publicly available due to privacy concern and patient confidentiality but are available from the corresponding author on reasonable request.

## Abbreviations

AVPD: Atrio-ventricular plane displacement
CMR: Cardiovascular magnetic resonance imaging
EDV: End-diastolic volume
EF: Ejection fraction
ESV: End-systolic volume
GLS: Global longitudinal strain
LAD: Left anterior descending coronary artery
LCx: left circumflex coronary artery
LVM: Left ventricular mass
MAPSE: Mitral annular plane systolic excursion
MI: Myocardial infarction
RCA: Right coronary artery
STEMI: ST-elevation myocardial infarction
WT: Wall thickening
